# *“Cooperation between physicians and physios fosters trust you know”:* a qualitative study exploring patients’ experience with first-contact physiotherapy for low back pain in French primary care

**DOI:** 10.1186/s12875-024-02302-x

**Published:** 2024-02-23

**Authors:** Amélie Kechichian, Dylan Pommier, Léo Druart, Véronique Lowry, Nicolas Pinsault, François Desmeules

**Affiliations:** 1 University Grenoble-Alpes, CNRS, UMR 5525, VetAgro Sup, Grenoble INP, TIMC, Grenoble, France; 2 Department of Physiotherapy, University Grenoble-Alpes, 175 Avenue Centrale, Saint-Martin d’Hères, Grenoble, 38400 France; 3 Maisonneuve-Rosemont Hospital Research Center, University of Montreal Affiliated Research Center, Montreal, QC Canada; 4 School of Rehabilitation, Faculty of Medicine, University of Montreal, Montreal, QC Canada

**Keywords:** First-contact physiotherapist, Medical act delegation, Patients’ experience and perceptions, Qualitative study, Semi-structured interviews, Thematic analysis, Advanced practice physiotherapy

## Abstract

**Background:**

Physiotherapists working in collaboration with family physicians in French multidisciplinary primary healthcare clinics are now able to manage acute low back pain patients as first-contact practitioners in advanced practice roles. This includes medical act delegation such as making a medical diagnosis and prescribing medication. The aim of this study is to explore patients’ experience and perceptions when attending a first-contact physiotherapist (FCP) in an advanced practice collaborative primary care model for acute low back pain (LBP).

**Methods:**

A qualitative study using semi-structured interviews was conducted. Patients that consulted a FCP for acute LBP care in new collaborative model were included. Interviews were transcribed verbatim and inductive thematic analysis was performed to generate themes related to patients’ experience and perceptions.

**Results:**

Ten patients were interviewed (3 women, 7 men; mean age 36.5 ± 9.63 years). All LBP participants experienced important level of pain and disability. Four overarching themes related to patients’ experience with the new FCP model were formalized: 1) *“Going to see a physiotherapist who specializes in painful movements, well that makes sense to me”,* 2) *“Physiotherapist offered to give me exercises to do at home to relieve the back pain”*, 3) *“I went there feeling confident”*, 4) *“The physiotherapist can do more than just send you to see more appropriate people”*. Participants highlighted the need to receive timely and high-quality care and were receptive with being autonomously managed by a FCP. Overall, patients’ experiences with FCP model of care were positive. Participants were highly confident in the FCP’s ability to perform delegated medical tasks including making a medical diagnosis and prescribing oral medication such as analgesic drugs. Patients felt that a greater expansion of FCPs’ scope of practice was needed to improve the model.

**Conclusion:**

Findings from this study can inform the implementation of FCP in countries where patients are not typically granted FCP by underlining that patients are favourable towards the advance practice model as such models support timely and high-quality care. Further research is needed to better determine the future advance practice physiotherapists’ scope of practice in French primary and secondary care settings**.**

**Supplementary Information:**

The online version contains supplementary material available at 10.1186/s12875-024-02302-x.

## Background

Musculoskeletal disorders (MSKD) are increasingly prevalent and represent the leading cause of long-term pain and physical disability in the general population [1–3]. Among MSKD, low back pain (LBP) is the most prevalent condition, affecting hundreds of millions of people worldwide [4–6]. More specifically, LBP is the second most common reason for seeking a family practice consultation in France [7]. However, the increasing and excessive workload of French family physicians leads to significant delays in accessing care for non-urgent MSKD such as LBP [8, 9]. Considering the aging of the population and the increasing demand for MSKD care, this crisis is expected to rise [10, 11].

Evidence shows that increased wait times for LBP care lead to poorer prognosis in terms of patients’ pain and disability [12]. Furthermore, it is crucial to identify psychosocial risk factors early, as they contribute to a higher risk of developing persistent pain [5]. Persistent LBP can have a significant impact on patients’ general health, overall quality of life and is associated with increasing healthcare demand and costs [5].

To face the challenge of accessing primary care services, new models of collaborative care are emerging worldwide [13–15]. International data suggests that integrating trained physiotherapists in collaborative healthcare teams to care for MSKD patients can contribute to alleviating physician workload pressure, improve patient-related clinical outcomes and reduce time required to access care [16–18]. In these models, physiotherapists work as first-contact practitioners in advanced practice roles [19–22]. First-contact physiotherapists (FCP) are able to receive patients without medical referral and to provide traditionally medical acts such as triage, diagnosis or medications and imaging prescriptions [18, 23]. While they are mostly developed in secondary care or emergency settings, these models are internationally emerging in primary care where FCPs work as substitutes to family physicians when caring for patients presenting non-urgent MSKD [20, 24–26].

In France, patients are traditionally referred to physiotherapists by a family physician. However, considering primary care services’ overcrowding, a new model has been recently implemented to allow task shifting from family physicians to FCPs working in multidisciplinary primary healthcare teams when caring for patients with acute LBP [27]. Eligible patients may directly consult a FCP instead of a family physician. As a result, the usual scope of practice of French physiotherapists is expanded, allowing them to make a medical diagnosis as well as prescribe analgesic medications (paracetamol, oral non-steroidal anti-inflammatory drugs and a proton pump inhibitor), deliver medical sick leave certificates and refer patients to family physician or additional physiotherapy treatments [27]. Although not formally defined as an advanced practice model by French authorities, this model entitled *“cooperation protocols”* conforms to the globally accepted definition of advanced practice physiotherapy [28, 29]. In order to evaluate the efficacy of this new model of care, a pragmatic cluster randomized controlled trial (RCT) is being conducted by our team *(ClinicalTrials.gov NCT05200533).*

There is growing interest in assessing patients’ experiences with healthcare as patients’ opinion is now recognized as a decisive aspect of care quality [30]. Evidence suggests that better patient experience with healthcare is associated with higher levels of adherence to recommended treatments processes, clinical outcomes and decreased use of healthcare resources [30, 31]. Understanding patients’ experiences of care may help improve quality of healthcare. Patients’ experience, defined as *“the sum of all interactions shaped by an organization’s culture that influence patient perceptions across the continuum of care”* is a measure of patient centredness with care, which is one of the six recognized healthcare quality objectives [32, 33]. Previous studies have shown patients favourably perceived new models of care, including advanced roles of FCPs in the management of MSKD [34–36]. Patients reported benefits of the new advanced practice physiotherapy models of care in terms of shorter wait times and being seen by a specialist who listened and involved them in their care management [26, 34, 35]. Studies have shown that extending physiotherapists’ scope of practice may facilitate patient self-management and breakdown role boundaries that contribute to professional hierarchy [26]. The recently implemented FCP advanced practice model in France may therefore represent a shift in patients’ perception of the care they receive.

The RCT conducted by our team includes a quantitative measure of patients’ satisfaction with the care they received using a standardized questionnaire. Exploring patients’ experiences qualitatively could, however, allow a broader and more representative assessment of patients’ perception with the care they received [30, 32]. This study aimed to explore the experiences and perceptions of patients attending FCP-led care for LBP in French multidisciplinary primary healthcare clinics.

## Methods

### Design

The data in this qualitative study were collected through semi-structured interviews with patients aiming to explore their experience of a new FCP model for the management of acute LBP in primary care settings. This qualitative study was nested within a cluster randomized controlled trial aiming to assess the model’s efficacy in six multidisciplinary healthcare clinics in France. To ensure credibility and transparency of the findings, the *Consolidated Criteria for Reporting Qualitative Research (COREQ)* tool added to recommendations on qualitative study methods were used in the designing and reporting of the study [37–39].

### FCP model of care

Nine physiotherapists have been recruited and trained to deliver care for acute LBP patients within the FCP advanced practice model. This model is described as a protocol-based care in which patients aged 20–55 years that have been suffering from LBP since less than 4 weeks can consult the FCP directly without having to see the physician first [27]. The objectives of the model are to give physicians more time to care for patients with more serious or complex pathologies, to reduce delays and improve quality of care. FCPs and physicians undergo a shared 10-h training before the implementation of the model [27].

During the assessment, the FCP identify patients requiring further medical care by screening for red flags during medical history and physical examination. The FCP also screen for yellow flags to identify psychosocial risk factors. FCP formulate a diagnosis and an intervention plan including therapeutic education, active exercise rehabilitation and physical activity promotion. If relevant, the FCP can prescribe analgesic medication, non-steroidal anti-inflammatory drugs and sick leave certificates of up to five days. FCP is also able to recommend further referral to physiotherapy follow-up sessions. Family physicians can be reached by phone at any time during the consultation to answer questions and ensure that the patients received any necessary medical care.

### Settings, recruitment and eligibility

Patients were first identified by a medical assistant when they set up an appointment with the family physician for a new onset of LBP. In the three primary care clinics randomized and allocated to the experimental arm, patients were proposed to consult the FCP. Patients who agreed to participate were independently assessed and managed by the FCP.

Each patient included in the RCT consented to be contacted for a follow-up appointment. They were contacted by phone by a research assistant in the weeks following the initial FCP consultation and were proposed to participate to the qualitative study. If they agreed to participate, the consent form was explained, sent to them by email and was signed by the participant before the interview. An appointment was suggested following the patients’ preferences. Individual semi-structured interviews were completed either virtually or over the phone.

Inclusion criteria were: 1- Patients aged from 20- to 55-year-old, 2- receiving care from FCP for a new onset of LBP in one of the three primary care clinics allocated to the experimental arm of the RCT, 2- able to understand and speak French, 3- agree to participate in the qualitative study. Inclusion criteria were defined according to the French legislative text of the *“cooperation protocol”.* Unavailability to fix an appointment for the interview was an exclusion criteria.

### Data collection

Following a review of relevant literature, an initial interview guide was developed for this study. It was written by one author (D.P.) and reviewed by a second author (A.K.) [34, 35]. Both authors are physiotherapists and the second author had previous experience with qualitative research. The interview guide aimed to explore patients' experiences and perspectives of the FCP-led advance practice model of care. The guide had six main questions designed to explore the patients’ experience of care, acceptability, satisfaction, perceived value of the FCP role and suggestions on how to enhance the FCP model of care (see Supplementary file). This interview guide was tested with one patient and no adaptations were needed following this first interview. This interview was not included in the analysis. The interviews were audio-recorded and transcripts were anonymized. Before the interviews, participants were informed that the interviewer was a physiotherapist. They were also assured that he did not work in the primary care setting where they received care and that they could speak freely of their experience. The researcher used active listening techniques and had formal education in qualitative methodology [38]. To profile interviewees, patient demographic characteristics collected in the RCT were used (age, gender, professional status, previous care management for low back pain and previous experience with physiotherapy).

### Data analysis

The sampling strategy aimed to reach thematic saturation, which refers to the point at which no new thematic information is gathered from participants [38]. Interviews were transcribed gradually during the interviewing process to help identify thematic saturation. Given the topic of our study and the relative homogeneity of the sample due to the specific inclusion criteria of the RCT (patients aged from 20 to 55 years old suffering from a new onset of acute low back pain) and considering prior similar studies, we anticipated that 8 to 15 participants could be included [34].

Based on the Braun and Clarke process, an inductive thematic analysis comprising five steps: pre-analysis, coding, categorization, refining and interpreting was conducted [39, 40]. Pre-analysis and coding were performed by two authors, the interviewer (D.P) and a physiotherapist researcher (A.K). Both researchers familiarized themselves with the transcripts and set up an initial code set for the first two interviews independently using an iterative approach. Discrepancies between the two code sets were reviewed and a final code set was decided. The final code set was then applied (by D.P.) to the other nine interviews. Themes were identified and refined following ongoing critical discussion between researchers (D.P. and A.K.) until consensus was reached. The online software QCAmap was used to aid during the coding phase.

## Results

### Participants’ description

Interviews were conducted with ten patients of which three were women and seven were men (aged 36.5 ± 9.63). Except for two participants, all of them had already received usual medical care for a previous onset of LBP. Only three participants had never experienced physiotherapy care before the FCP consultation and two participants had already consulted a physiotherapist for back-related symptoms. Participants’ characteristics are presented in Table 1.

**Table 1:** Participants’ characteristics (*n* = 10)

Patient	Gender	Age	Professional status	Previous care management for low back pain	Previous experience with physiotherapy (reason)
[P1]	M	22	Employed	Y	Y (back)
[P2]	F	53	Employed	Y	Y (shoulder)
[P3]	M	38	Employed	Y	Y (back)
[P4]	M	43	Employed	Y	N
[P5]	M	32	Employed	N	Y (ankle)
[P6]	M	42	Employed	Y	N
[P7]	F	38	Employed	Y	Y (foot)
[P8]	F	31	Unemployed	Y	Y (knee)
[P9]	M	43	Employed	Y	Y (knee)
[P10]	M	23	Student	N	N

### Thematic analysis

Four overarching themes related to patients’ experience with the new FCP model were formalized from our thematic analysis: 1) *Going to see a physiotherapist who specializes in painful movements, well that makes sense to me*, 2) *The physiotherapist offered to give me exercises to do at home to relieve the back pain*, 3*) I went there feeling confident*, 4) *The physiotherapist can do more than just send you to see more appropriate people.*

Main themes and subthemes are presented in Table 2.

**Table 2:** Thematic analysis main themes and subthemes

Main themes	Subthemes
*“Going to see a physiotherapist who specializes in painful movements, well that makes sense to me”*	• Patients had important sudden pain and disability • Patients were expecting to be quickly managed • The FCP model made sense for patients
*“The physiotherapist offered to give me exercises to do at home to relieve the back pain”*	• Rapid access prevented complications • Positive informed experience was offered for the evaluation and care plan
*“I went there feeling confident”*	• Physiotherapists were perceived as competent first-contact practitioners that may replace physicians • Physiotherapists had the competences to make a valid diagnosis, prescribe medication and sick leaves • Physiotherapists gave thorough education and explanations and listened to patients • The FCP model supported interprofessional collaboration
*“The physiotherapist can do more than just send you to see more appropriate people”*	• Clearer definition of the FCP advanced practice roles and model was needed • Physiotherapists should be allowed to prescribe longer sick leave certificates and additional medication • The model should include additional physiotherapy follow-up sessions

### Theme 1: *“Going to see a physiotherapist who specializes in painful movements, well that makes sense to me”*

#### Patients had important sudden pain and disability

All participants experienced severe levels of pain and associated disability when they sought care for their new LBP onset, as one participant mentioned: *“It was completely impossible for me to bend forward. Basically, I was blocked.”* [P2]. For most of them, symptoms suddenly appeared and could be associated with unusual professional or mechanical physical constraints: *“It was very busy in terms of my workload»* [P2], *“I had to carry packages, they were much too heavy and it was much too repetitive.”* [P2]. Some participants identified that they thought *“there also was a big psycho-emotional aspect to it.”* [P8] that could have played a role in the perception of their symptoms. One participant stated that she *“was feeling really stressed out.”* [P2]. Moreover, beliefs and catastrophization regarding back pain were found for some of the participants. For example, a participant mentioned: “*Then, I said to myself, “Well, I really need to see a doctor, isn’t there something serious going on?» (…) And you know I started thinking the worst. Could it be the kidneys? Could it be the liver?”* [P6].

#### Patients were expecting to be quickly managed

By seeking care and booking a visit in their primary healthcare clinic, most of the participants expected to be quickly managed to relieve their pain as expressed by [P2] saying she “*only wanted one thing, to be taken care of as quickly as possible*”; or by [P8] stating she *“was in a lot of pain and I needed pain relief first.”* Some participants consulted “*to be put on medical leave because I couldn’t work anymore*” [P2] while others noted they rather needed to be reassured, as mentioned by [P6]: “*I usually want to be reassured by doctors and other care providers*”.

#### The FCP model of care made sense for patients

Most patients were not surprised to be directly and autonomously cared for by a FCP: *“I thought it made sense to send me to a physio to check my back”* [P2]. One participant believed that *“the doctor and the physiotherapist have the same role for this pathology [low back pain]”* [P3]. However, for other participants, *“I think physios are more specialized, you know, for these types of problems with joints, than family physicians are”* [P4].

According to some participants, this new model could help alleviate family physicians’ workload “*if our family physician has a pretty busy schedule, well [the FCP model] allows him to unload some of his cases*” [P1]; and *“it avoids taking the place of someone else with an emergency in fact*.” [P6]. Participants reported that this model also *“helps you get that first appointment, going to see a physio directly”* [P5] and that *“it highlights the skills that a physio can have”* [P7] such as expertise in the management of patients with LBP.

### Theme 2: *“The physiotherapist offered to give me exercises to do at home to relieve the back pain”*

#### Rapid access prevented complications

Participants reported being received shortly after calling for an appointment, either *“on the same day”* [P1] [P4] [P8] or *“on the next day”* [P2]. Participants reported access to care to be timelier than what they expected with primary care services: *“In the end, I was taken care of very quickly”* [P3], [P7]; *“It’s a time saver”* [P3] [P4] [P6]. Participants stated that early access to care was important *“especially for back pain, it’s really important”* [P4]. They thought that *“it helped avoid a lot of problems later on actually. Because I would have made it worse instead of fixing the problem”* [P7]. One participant felt reassured by accessing physiotherapy care earlier as* “it removes a stress factor in the pathway to rehabilitation, which can be difficult, by getting you where you actually really start to work on the core of the problem basically”* [P7].

#### Positive informed experience was offered for the evaluation and care plan

First, patients described that *“the physio told me all about the different steps that I was going through, what care I needed and what was expected from the treatment itself”* [P9]. They reported they experienced with their initial visit and care plan *“a pretty complete exam and an anti-inflammatory prescription which clearly helped. Recommendations for a few exercises and a few sessions with a physio”* [P8] and were provided with *“a few days off work to rest you know”* [P2] as well as *“exercises to do at home to relieve the back pain”* [P9]. Patients reported satisfaction with the care they received and found it to be a *“positive experience”* [P2].

### Theme 3: *“I went there feeling confident”*

#### Physiotherapists were perceived as competent first-contact practitioners that may replace physicians

Participants perceived their PT as an *“expert”* [P2] in the assessment and treatment of low back disorders and thus were confident in his or her ability to manage them as a primary-contact practitioner. P1 felt reassured as* “seeing someone who does this every day, that has this type of clientele with lumbago problems, so who is used to dealing with that, of course it’s comforting.”*

Participants were receptive to being managed by a FCP in order to save time and benefit from adequate and early advice: *“For some things, there’s not really any need to go through the doctor […] it’s a waste of time”* [P9]. One participant thought that “*the family doctor could have told me what I had. But he would have said “this is what you have. Go see a physio!” and meanwhile the physio would have told me what I had again, but given me advice straight away*” [P5]. Some participants even suggested that FCP may sometimes be more competent than family physicians in the primary management of musculoskeletal disorders as *“it’s your specialty, it’s your job to analyze painful movements. Which is not always obvious for a doctor you know”* [P10].

However, referral to another professional when unable to manage appropriately was expected: *“That’s what I expect from a health professional. He knows what to do, good. He doesn’t know, he sends you to someone else”* [P9]. Some of them added that* “later on, if it had gotten worse, maybe I would have asked to see a doctor”* [P2].

#### Physiotherapists had the competences to make a valid diagnosis, prescribe medication and sick leaves

Participants reported being *“confident”* in the FCP ability to diagnose non-specific acute LBP. This high level of confidence could be explained by the perceived ability of the FCP to adequately screen for signs and symptoms of serious pathologies and precisely identify the disorder they suffered from: *“I described everything that happened and she didn’t find anything to worry about, anything in particular, and I think she was able to assess my situation and that it wasn’t so bad (…) So, that was comforting”* [P6].

Timely assessment and diagnosis were highlighted since *“very, very quickly he was able to make the diagnosis”* [P9]; “*Basically, the diagnosis he gave me was really about where the pain I was feeling was focused*” [P7]. Explanations given by FCPs regarding their symptoms strengthened the confidence level participants had in the diagnosis process *“also, he explained what I had really well (…). Finally, the why and the how. And where it was coming from”* [P3]. One participant strongly trusted FCP’s competence for diagnosis as he said: *“if I go to a family doctor and he says “It’s lumbago” and when I get to the physio he says “well no, it’s not lumbago, it’s more something like this.” I would tend to think that the physio is right and not the family physician”* [P5].

FCPs were also viewed as *“competent”* [P7] to prescribe appropriate medication for LBP: *“I think it’s actually good that they are allowed to prescribe level I analgesics”* [P7]. This confidence level could be explained because *“it’s still within the medical framework” and “we’re not talking about really, really strong medication also”* [P8].

Regarding sick leave certificates, some participants considered that “*a physiotherapist can say just as well as a doctor if we are able to work or not*” [P5]. For certificate prescription, they reported that *“whether he is the one who does it because now he’s allowed, or the doctor, it doesn’t change anything whatsoever”* [P9].

#### Physiotherapists gave thorough education and explanations and listened to patients

Participants seemed to consider they received accurate and complete information from the FCP: “*She gave me detailed explanations about the exercises I needed to do”* [P1]; *“I got the answers to all my questions with the physio”* [P3]. They were satisfied with the consultation duration since *“one of the major benefits is to have an appointment with someone […] who can take more time to examine you”* [P1].

Participants thought that *“these are people who are really listening to us”* [P2]. Active listening and patient-centred approach used by FCPs enabled them to feel involved in their own management: *“She still asked for my opinion”* [P8]; *“She asked me ‘Let’s be clear, do you feel ready to go to work?’*” [P6].

#### The new FCP model of care supported interprofessional collaboration

Participants stated that *“one of the benefits of this care model is that there is a kind of mutual cooperation between the different departments and that the doctors and the physios work together”* [P2]. They felt that *“there were real discussions still going on behind the scenes with the doctor, and that it was not unsupervised you know”* [P8]. According to them, *“it’s the way the care center where they work is set up that makes it possible for them to work together and not be afraid to call someone to say: ‘I have doubts, a problem, what do you recommend? How do we do this?’”* [P10].

Existing interprofessional collaboration between FCPs and family physicians could have emphasized the level of confidence participants had towards the model of care as *« cooperation between physicians and physios fosters trust you know”* [P8].

### Theme 4: *“The physiotherapist can do more than just send you to see more appropriate people”*

Some participants had suggestions of improvements on the existing model based on the limitations they experienced and suggested expanding the advanced practice roles and the autonomy of the FCPs.

### Clearer definition of the new FCP advanced practice roles and model was needed

Several participants felt a lack of information given by the administrative staff about the role and competencies of the FCP before the consultation. They suggested “*maybe, from the start, explain to me what will happen with the physio because they just told me to go see a physio*” [P10] or *“to explain the role of the physio and that there’s more to it than just the ‘basic physio diagnosis’, and that you have a global approach and that you know, hmm, you can make a diagnosis”* [P8]. For participants, this information is important to reinforce the confidence level they had regarding the ability of the professional to safely manage them, especially when they never experienced physiotherapy care before.

### Physiotherapists should be allowed to prescribe longer sick leave certificate and additional medication

Some participants suggested that FCPs *“should be able to renew time off work at least once”* [P5] to avoid a new consultation with family physicians as *“I had to go back to the physician to renew time off work”* [P4]. Regarding medication prescriptions, two participants mentioned that FCPs should be allowed to prescribe *“something a little bit stronger”* [P4] as they did not get the medication they needed because the FCP was not allowed to prescribe it.

### The model should include additional physiotherapy follow-up sessions

Participants reported that the FCP model did not include enough physiotherapy follow-up sessions as they *“had to request a new prescription to the physician just to continue physiotherapy”* [P7] because *“with the protocol they had, they were allowed three sessions without a medical opinion but in the end, they still needed a referral”* [P9]. One participant suggested that FCPs should be allowed to prescribe five to ten outpatient physiotherapy sessions. According to her *“it doesn’t represent a huge risk for the physio”* [P7]. One participant, however, mentioned that *“the problem is that she prescribed 3 sessions with a physio and when I called to get an appointment, I couldn’t find one, I gave up… So, I didn’t go do the 3 sessions”* [P6].

## Discussion

This qualitative study explored the experience and perceptions of patients with a new primary care model of FCP advanced practice for LBP. The identified themes were related to patients’ acceptability of the new model, experience with the care they received, perceptions of FCPs’ skills and enhancement perspectives of the new model of care.

One of the key findings of our study was that patients were very receptive to consult FCPs instead of family physicians. This is an important finding since in France, patients are traditionally referred to physiotherapists by family physicians. Patients highlighted the given opportunity to use physiotherapists’ skills in the management of MSKD and thus enable physicians to dedicate their time for patients with more complex or serious pathologies. This finding is consistent with another study exploring how patients perceived seeing a FCP instead of a physician in Australian emergency departments [35].

Experiences of the FCP model of care were positive and patients were largely impressed by short wait times they underwent for initial assessment. An earlier study has shown that patients’ expectations on long wait times associated with family physicians’ consultations increased the acceptability of advanced practice physiotherapy management [26]. However, faster management could have been influenced by the experimental context of the pragmatic cluster RCT which was running in the different healthcare clinics where participants of this study were recruited. In order to adequately evaluate the impact of the FCP model on patients’ clinical outcomes and healthcare use, investigators of the RCT were asked to receive patients in a timely manner. Short wait times could be a secondary benefit of the experimental study and did not fully represent the usual clinical practice. When asking how to enhance the new model, some participants underlined the need to facilitate physiotherapy follow-ups within the first care setting as they were unable to access care elsewhere. This finding shows that access to physiotherapy care is unequal when considering FCP model compared to usual physiotherapy care.

Another key finding is that participants expressed high levels of confidence in the FCP’s ability to independently diagnose and manage their acute LBP. This could be explained because participants felt physiotherapists had better expertise than physicians in the management of MSKD due to their specialist training and knowledge. This is a significant result showing that respondents were receptive to the evolution of the gatekeeper positioning of family physicians regarding access to primary care services and diagnosis of MSKD. These results are concordant with views from French physiotherapists and physicians who reported the new FCP model to be highly acceptable [41].

Overall, patients were confident with the FCP’s competency to perform medical tasks such as prescribing medication and sick leaves. This may be explained by the low risk participants associated with performing these tasks. This seemed especially the case with the prescription of low-class analgesic medication. Patients perceived an added value with benefiting from a *“one-stop”* consultation that met their prior expectations. The perceived knowledge and skills of FCPs regarding MSKD management also seemed to reinforce patients’ acceptance with seeing a PT in a role traditionally done by family physicians. However, some of them added that in case of persistent symptoms or if the FCP has any doubt on their condition, they would expect to be referred to the family physician. This finding pointed out that FCPs should be able to adequately determine when the patient’s condition exceeds the scope of their competencies he has been trained for in this model.

Professional competencies of FCPs were recognized and valued by participants of our study. They mainly reported that FCPs listened to them and had enough time to spend with them. FCPs provided patient-centred care that enabled participants to be actively involved in their care management and contribute to reducing the risk of developing chronic conditions [42, 43]. Similar results were found in a previous review about patients’ views on advanced practice physiotherapists [26]. Psychosocial risk factors were identified for most of the participants when they described the initial symptoms that encouraged them to attend for a medical appointment. If not adequately considered, these factors can lead to poorer clinical outcomes and persistent level of pain and disability [44, 45]. The need to adequately address patients’ psychosocial risk factors legitimates the positioning of physiotherapists as primary-contact practitioners for the management of LBP patients [12, 46, 47].

Pre-existing interprofessional collaboration between family physicians and FCPs working in the same multidisciplinary healthcare clinics seemed to favour the acceptance and confidence participants had in the model. Indeed, appropriate physician referral in case of suspected non-MSKD condition or that required medical review is easier when professionals already achieved a good mutual knowledge and are used to communicate efficiently [48–50].

Regarding possible improvements of the model, participants’ suggestions mostly expanding FCPs’ role and/or scope of practice in the model. Participants would have liked that FCPs could be allowed to prescribe stronger analgesic medications, longer sick leave certificates and refer them to additional physiotherapy sessions all so that they did not need to get back to the physician. The process for such a change is expected to be long, as the proposed FCP model is already considered as a major change in the French PT practice by professionals and authorities. Consideration of these suggestions could, however, enable a better tailoring of the model by preventing unnecessary or inefficient use of family physicians time and expertise.

Another important finding is that patients would have liked to be better informed about FCPs’ roles, competencies and skills in this new advanced practice model, especially when they had never consulted a physiotherapist before. This result is consistent with previous studies showing that patients’ education on physiotherapists’ competencies, skills and related training is a determining factor that influence patients’ acceptability of advanced practice models of care in physiotherapy [26]. This review also highlighted the importance of the receptionist’s role in increasing patient understanding and awareness of advanced practice physiotherapy models [26].

The acceptance of non-medical health professionals working in roles traditionally performed by medical professions is consistent with other studies involving nurse practitioners and advanced practice physiotherapists conducted in different countries and care settings [34, 35, 51]. These models are a promising strategy to address the physician workforce shortage while offering efficient and high quality care [23]. Further research is, however, needed to better determine the acts and interventions patients found acceptable for advanced practice physiotherapists’ delivery and to explore related practitioners’ self-efficacy feeling [26].

### Strengths and limitations

The present study has major strengths. Participants were recruited over three different primary care settings. Inductive analysis was conducted to ensure that the findings emerged from the data and not from the researcher’s perception. Findings of this study were consistent with other studies exploring patients’ perspectives with advanced practice physiotherapy models and with the results of a previous acceptability study on this FCP model conducted by our team [26, 35, 41].

Some limitations should, however, be considered. The FCP model had only been recently implemented in France and only a few patients had benefited from this model. Thus, the limited sample of participants we could target for this qualitative study did not enable us to recruit only participants that have been recently managed by physiotherapists. Patients’ experiences and perceptions collected in the interviews depended on how precisely patients remembered what happened during the consultation. Their perceptions could have been influenced by the positive or negative evolution of their symptoms over the time. Patients’ discourse could also have been influenced by knowing that the research assistant who conducted the interview was a physiotherapist student, even if he mentioned that he did not work within the healthcare settings where they received treatment. Then, we did not collect information about patients’ perception of the family physicians consultations before the study. We could not strongly support whether patients experienced better service with the new model. Last, the experimental context in which the FCP model was set up could have been not fully representative of the usual clinical practice, even if the RCT was designed in a pragmatic perspective. Patients’ perceptions could have been positively influenced by this context, has shown, for example, by potentially shorter waiting times they experienced.

## Conclusion

According to the participants, the recognized competencies and expertise of physiotherapists in the musculoskeletal field placed them as suitable alternatives to family physicians for managing acute low back pain in this new model of care. Participants highlighted the need to receive high-quality and timely care while being very receptive with physiotherapists being primary-contact practitioners. Participants expressed high confidence in the competency of physiotherapists to perform medical tasks including diagnosing and prescribing medication as long as the physiotherapist refers them back to a physician if there were any suspicion indicating a more complex or a serious condition or if their condition was not resolving. Existing interprofessionnal collaboration between physiotherapists and family physicians appears to increase patients’ confidence level in this innovative healthcare pathway.

Further research is, however, needed to encourage formal recognition of advanced practice physiotherapy by the French authorities and to better determine the future roles and scope of practice of French advanced practice physiotherapists in primary and secondary care settings**.**

### Supplementary Information

**Supplementary Materials 1.**

## Data Availability

The datasets used and analysed during the current study are available from the corresponding author on reasonable request.

## Abbreviations

MSKD: Musculoskeletal disorder
LBP: Low back pain
FCP: First-contact physiotherapist
RCT: Randomized controlled trial
