# Supplementary material for: “Cooperation between physicians and physios fosters trust you know”: a qualitative study exploring patients’ experience with first-contact physiotherapy for low back pain in French primary care
Source: BMC Prim Care. 2024 Feb 23;25:69. doi: 10.1186/s12875-024-02302-x (PMC10885482; doi:10.1186/s12875-024-02302-x)
Supplement: Supplementary file 1 — Supplementary Materials 1. [file 12875_2024_2302_MOESM1_ESM.docx]

| **Topic** | **Key questions** | **Prompts** |
| --- | --- | --- |
| **Patients’ experience of care** | Can you describe the care you received for your low back pain problem? | Who have you seen for your low back pain problem?  What happened at those appointments?  Did you get any diagnosis, treatments, and advices? |
| **Acceptability and perceptions of the new model of care** | What did you expect form this appointment?  How did you feel about seeing a physiotherapist as first-contact practitioner? | How did this appointment compared to a physician’s appointment?  What makes it different or the same?  Are there any advantages or disadvantages? |
| **Perceptions of the physiotherapist’s skills** | In this new model of care, the physiotherapist perform traditional medical acts such as medication prescription, sick leave certificate delivery or physiotherapy referral.  How did you perceived physiotherapist’s skills for these medical acts? | How competent was the physiotherapist to manage you as first-contact practitioner?  What did you think about the physiotherapist’s skills to prescribe you adequate medication and to deliver a sick leave certificate?  How confident you were about the care you received from the physiotherapist? |
| **Satisfaction with care** | How satisfied you were with the care you received? | To what extent did the care and the treatment you received meet your expectations?  What did you like or not during the care you received? |
| **Benefits, limits and improvement perspectives of the model** | According to you, what are the benefits and limits of this new model of care for the patients?  How this model could be improved? | What could have improve your experience? |
| **Conclusion** | Is there anything else you would like to tell me about your experiences with the new model of care for you low back pain problem? | |

**Supplementary file : semi-structured interview guide**
